# Effectiveness of Soil, Foliar, and Seed Selenium Applications in Modulating Physio-Biochemical, and Yield Responses to Drought Stress in Vegetable Soybean (*Glycine max* L. Merrill)

**DOI:** 10.3390/plants14213261

**Published:** 2025-10-24

**Authors:** Masego Sekhurwane, Brigitta Tóth, Makoena Joyce Moloi

**Affiliations:** 1 Department of Plant Sciences-Botany Division, Faculty of Natural and Agricultural Sciences, University of the Free State (Bloemfontein Campus), 205 Nelson Mandela Drive, Park West, Bloemfontein 9301, South Africa; 2015224492@ufs4life.ac.za (M.S.); moloimj@ufs.ac.za (M.J.M.); 2 Institute of Food Science, Faculty of Agricultural and Food Sciences and Environmental Management, University of Debrecen, Böszörményi Str. 138, 4032 Debrecen, Hungary; 3 Institute of Engineering and Agricultural Sciences, University of Nyíregyháza, Sóstói Str. 31/b, 4400 Nyíregyháza, Hungary

**Keywords:** antioxidants, drought stress, photosynthesis, selenium, vegetable-type soybeans, yield

## Abstract

Drought stress severely affects the physio-biochemical processes and yield of nutritious crops like vegetable soybean (*Glycine max* L. Merrill), threatening global food security and emphasising the need for effective strategies to improve drought tolerance. This study, conducted under controlled conditions in a greenhouse, investigates the effects of three selenium application methods (seed priming, foliar spray, and soil application) on photosynthesis efficiency, relative water content (RWC), hydrogen peroxide (H_2_O_2_), antioxidative responses, and yield traits of two vegetable soybean cultivars, UVE14 (drought-tolerant) and UVE17 (drought-susceptible), under drought stress. Among the three Se application methods, soil application was the most effective in improving drought tolerance and yield performance in both cultivars. In UVE17 (drought-susceptible), soil application significantly increased the number of seeds per plant (SPP) and the number of pods per plant (PPP), while in UVE14 (drought-tolerant) SPP increased. Selenium foliar spray and seed priming treatments did not increase yield in drought-stressed UVE14, suggesting that they are unlikely to further enhance tolerance in drought-tolerant cultivars. For UVE17 under drought conditions, selenium soil application improved key physio-biochemical indicators of drought tolerance, including photosynthesis efficiency (total performance of photosystems I and II, total chlorophyll content, and stomatal conductance), water retention (RWC), and carotenoid content. These improved physio-biochemical responses directly impacted yield outcomes. Notably, RWC and total chlorophyll content at the pod-filling stage in drought-stressed UVE17 were positively correlated with an increase in PPP under selenium soil application. Selenium soil application stands out as the most effective method for enhancing drought tolerance in vegetable soybean, presenting a promising and practical solution for enhanced crop production under climate change.

## 1. Introduction

Africa has the highest number of undernourished people in the world, with sub-Saharan Africa accounting for 92% of this population [1,2]. To address this issue, there is a need to grow new, highly nutritious crops. One such crop is vegetable soybean (*Glycine max* L. Merr.), also called edamame, a type of soybean known for its large seeds. Vegetable soybean is an excellent source of protein, containing all essential amino acids, as well as being rich in oil, minerals, and dietary fibre [3,4]. Globally, its popularity as a nutritious snack and versatile ingredient is increasing, especially in Asia and Western countries, where there is a growing demand for plant-based protein options [5]. In Africa, vegetable soybean has been introduced as a promising crop to combat malnutrition in South Africa (SA) and 25 other African countries [2]. With its high nutritional value and adaptability to different growing conditions, vegetable soybean could play a big role in improving food security across the continent.

However, the successful cultivation of vegetable soybeans, particularly in regions prone to food insecurity, faces significant challenges due to environmental factors such as drought. While its nutritional benefits make it an excellent crop for combating malnutrition, vegetable soybean presents a challenge due to its substantial water needs throughout its developmental stages [4]. This is particularly problematic in SA, a semi-arid country with unpredictable weather patterns, especially in soybean-producing regions [6]. In 2015, SA declared a national state of disaster due to severe drought, resulting in economic losses exceeding 200 million US dollars and affecting over 2.7 million people [7]. Therefore, it is important to highlight how drought, a major environmental stress associated with climate change, affects the physiological and biochemical responses of crops like vegetable soybean, as these changes can significantly affect yield and, in turn, the farming industry [8].

Drought stress disrupts key physiological processes like photosynthesis, which in turn hinders crop growth, development, and yield [9,10,11,12,13]. Furthermore, drought stress triggers the production of reactive oxygen species (ROS), which, in excess, can be harmful to plants. These ROS damage lipids, proteins, and nucleic acids, leading to cell damage and plant death [14]. To combat such losses, efforts to breed drought-tolerant genotypes are ongoing, though this process can take time. Meanwhile, selenium has been found to help protect plants from various environmental stresses, offering an alternative approach to traditional breeding methods for improving plant resilience. This raises the question: Can selenium improve drought stress resilience in vegetable soybean to improve yield? While selenium is essential for both animals and humans, it is considered a beneficial element to plants because it helps enhance their tolerance to stress [15,16]. Recently, selenium has been suggested as a potential solution for improving drought tolerance in crops, including vegetable soybean [17,18]. According to Seleiman et al. [17], selenium can promote plant growth, reduce oxidative stress damage, enhance antioxidant responses, and regulate water balance during drought stress.

A range of selenium (Se) compounds, including traditional forms (microparticles, such as selenite and selenate) and selenium nanoparticles (SeNPs), have been studied for their ability to mitigate drought stress in plants [19,20,21]. Application of nano-selenium during the reproductive stage of soybean enhances drought tolerance by promoting biomass accumulation, maintaining water content, and increasing photosynthetic pigment levels, while simultaneously improving oxidative stress management and ultrastructural integrity [22]. Both selenium microparticles and nanoparticles enhance drought tolerance by enhancing antioxidant enzyme activities (e.g., SOD, CAT, and POD); increasing osmoprotectant levels (e.g., proline, glycine betaine); improving photosynthetic pigment content and water status, and reducing oxidative damage and membrane lipid peroxidation [22,23,24,25].

Selenium, whether applied through foliar sprays, seed soaking, or soil incorporation, has consistently demonstrated its ability to increase relative water content (RWC), stimulate antioxidative defence, and improve crop resilience under stress. Foliar spraying with selenium nanoparticles has been shown to enhance drought tolerance in tomato by altering gene expression, upregulating antioxidant systems, and stimulating secondary metabolism [26]. Similarly, both organic selenium and nanoselenium enhance drought resistance in pak choi by increasing photosynthetic capacity, maintaining water homeostasis, and promoting biomass accumulation through the up-regulation of metabolic pathways [27]. In wheat, selenium foliar application has been reported to increase RWC and enhance photosynthesis [19] while seed soaking reduced hydrogen peroxide (H_2_O_2_) accumulation [20]. Soil application has also been associated with improved growth and yield in wheat [21]. Additionally, foliar selenium application has been linked to enhanced growth parameters and yield in soybean [22]. However, in vegetable soybean, foliar application at the vegetative stage increased antioxidative enzyme activity but did not improve yield [18]. Moreover, de los Ángeles Sariñana-Navarrete et al. [23] demonstrated that seed priming in Jalapeño with low concentrations of sodium selenite or selenium nanoparticles stimulates early growth (germination, radicle and plumule development, and seedling vigor) and enhances antioxidant activity, resulting in seedlings with greater stress tolerance. However, higher concentrations may be phytotoxic, emphasising the need to optimise selenium form and dose for improved crop resilience [23].

Given these varying outcomes, evaluating the effectiveness of different selenium application methods is of practical importance. Such comparisons can help farmers to identify the most convenient and efficient method for their specific conditions. This study investigates how different selenium applications (through seed priming, soil drenching, and foliar spraying) affect the physio-biochemical responses and yield of two vegetable soybean cultivars, UVE17 (which is more sensitive to drought) and UVE14 (which is more drought-tolerant). Using two different cultivars allows for a comparison of their responses to selenium treatments under drought stress.

## 2. Results

### 2.1. Three-Way, Two-Way, and Main Effects of Selenium, Application Method, and Cultivar

**Figure 1:**
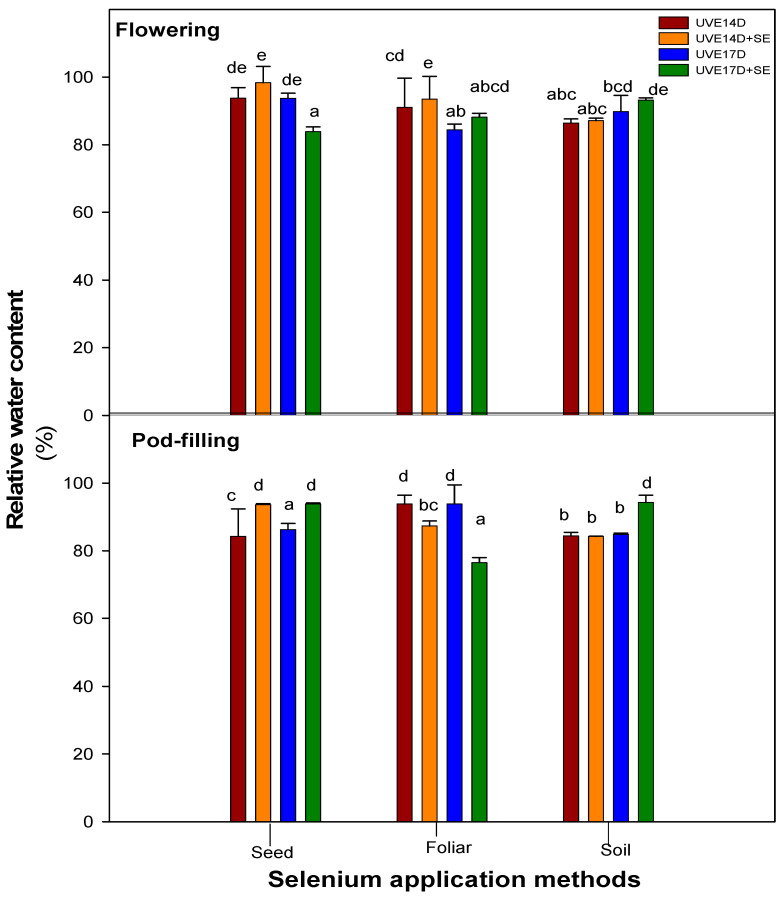
Relative water content (RWC) of drought-stressed vegetable soybean cultivars (UVE14 and UVE17) grown under the different selenium application methods (seed priming, foliar spray, soil drench) at the flowering and pod-filling stages. The values represent the mean ± SE (n = 3). Different letters on top of the bars indicate significant differences in the means within each selenium treatment at *p* ≤ 0.05. Fischer’s LSD post hoc test was used to determine the significance of differences between the averages (*p* < 0.05). Treatments with different letters are significantly different. If treatments share one or more letters, they are not statistically different. UVE14: drought-tolerant, UVE17: drought-susceptible, D: drought stress, D+Se: drought stress under selenium treatment.

**Figure 2:**
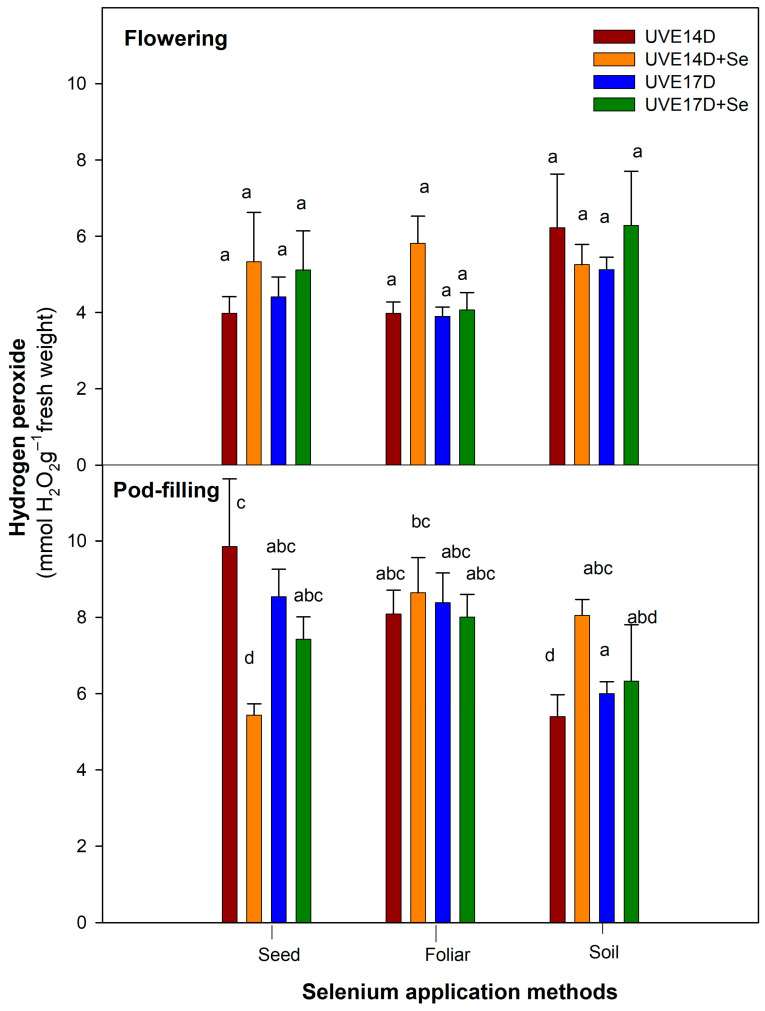
Hydrogen peroxide content of drought-stressed vegetable soybean cultivars (UVE14 and UVE17) grown under different selenium application methods (seed priming, foliar spray, soil drench) at the flowering and pod-filling stages. The values represent the mean ± SE (n = 3). Different letters on top of the bars indicate significant differences in the means within each selenium treatment at *p* ≤ 0.05. Fischer’s LSD post hoc test was used to determine the significance of differences between the averages. UVE14: drought-tolerant, UVE17: drought-susceptible, D: drought stress, D+Se: drought stress under selenium treatment.

**Figure 3:**
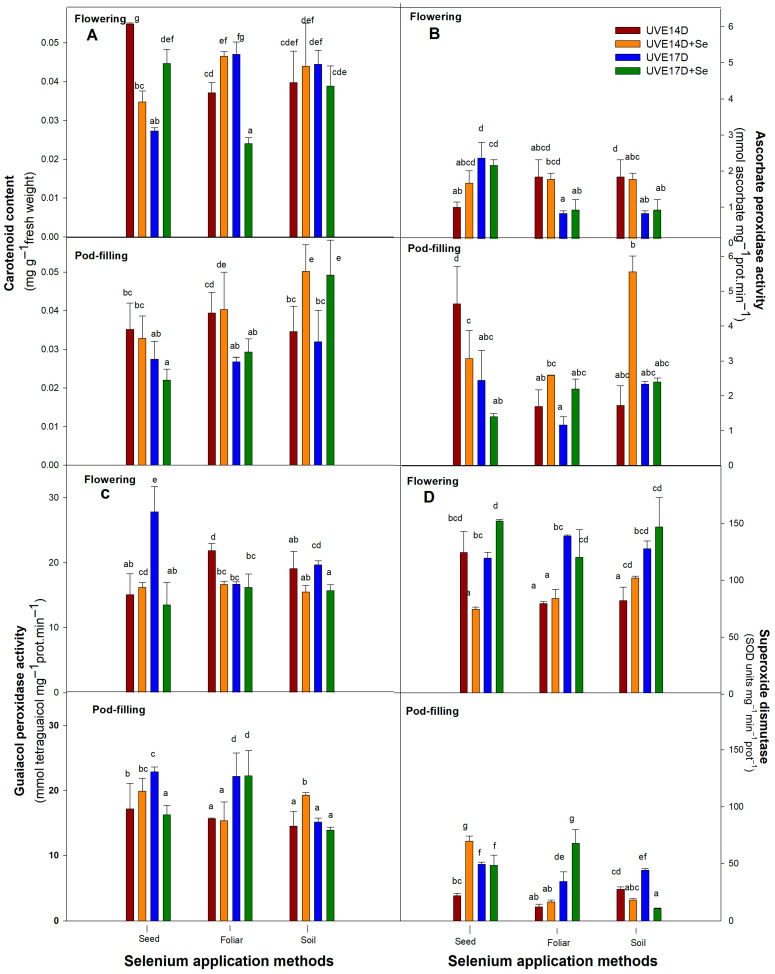
Carotenoid content (**A**), Ascorbate peroxidase activity (**B**), Guaiacol peroxidase activity (**C**), Superoxide dismutase activity (**D**) of drought-stressed vegetable soybean cultivars (UVE14 and UVE17) grown under different selenium application methods (seed priming, foliar spray, soil drench) at the flowering and pod-filling stages. The values represent the mean ± SE (n = 3). Fischer’s LSD post hoc test was used to determine the significance of differences between the averages. Different letters on top of the bars indicate significant differences in the means within each selenium treatment at *p* ≤ 0.05. UVE14: drought-tolerant, UVE17: drought-susceptible, D: drought stress, D+Se: drought stress under selenium treatment.

Table 1 presents the main effects of selenium (Se), application method (M), and cultivar (C), as well as their two-way (Se × M) and three-way (Se × M × C) interactions, on the physiological and biochemical parameters of two vegetable soybean cultivars (UVE14 and UVE17) at the flowering and pod-filling stages under drought stress (ANOVA). At the flowering stage, a significant three-way interaction (Se × M × C) was observed for total performance index (PI_total_), relative water content (RWC), and superoxide dismutase (SOD) (*p* ≤ 0.01), as well as total chlorophyll, carotenoid, stomatal conductance (g_s_), and guaiacol peroxidase (GPX) (*p* ≤ 0.001). At the pod-filling stage, RWC, GPX, SOD, hydrogen peroxide (H_2_O_2_) (*p* ≤ 0.001), ascorbate peroxidase (APX) (*p* ≤ 0.01) and carotenoid and g_s_ (*p* ≤ 0.05) were substantially influenced by the interaction. These significant interactions indicate that the effect of selenium application on these parameters varied between cultivars and depended on the method of application. Therefore, for further analysis, Fisher’s Least Significant Difference (LSD) test was performed to explain how each cultivar performed when selenium was applied under different application methods (data presented in Table 1 and Table 2 and Figure 1, Figure 2 and Figure 3).

Parameters that exhibited non-significant three-way interactions (above) indicated that their responses to selenium and application method were independent of cultivar. Therefore, in this case, the interpretation was based on the corresponding two-way interactions (Se × M). However, at the flowering stage, there was still a high number of non-significant physio-biochemical parameters, which included the ratio of variable fluorescence to maximum fluorescence (Fv/Fm), performance index absorbance (PIabs), heat dissipated per reaction centre (RC) (DIo/RC), absorption per RC (ABS/RC), electron flux per RC (ETo/RC), chlorophyll *a*, chlorophyll *b*, APX, electrolyte leakage (EL), and H_2_O_2_. At the pod-filling stage, PI_total_ was also non-significant in addition to the parameters that were non-significant at flowering. This suggests that, overall, the physiological and biochemical responses were largely consistent across application methods, indicating that selenium effects were independent of the method of application and cultivar. The only parameters showing a significant interaction between selenium × application method and not already significant in the three-way interaction were chlorophyll *b* and total chlorophyll (*p* ≤ 0.05) at the pod-filling stage. This suggests that chlorophyll synthesis and stability at this stage were influenced by both selenium availability and its method of application (Table 1). To identify specific treatment differences, these means were further separated using Fisher’s Least Significant Difference (LSD) test. The post hoc analysis provided a detailed comparison of treatment combinations contributing to the overall significance. Table 2 presents the mean separation results, highlighting significant differences (*p* ≤ 0.05) in chlorophyll *b* and total chlorophyll content during pod-filling. The table shows the two-way interaction between selenium concentration and application method on the physiological and biochemical parameters of drought-stressed edamame, excluding cultivar effects at the pod-filling stage. Compared to the drought-stressed control without selenium, seed dressing reduced total chlorophyll content by 21%, whereas foliar and soil drench applications increased it by 38% and 36%, respectively. The similar responses observed under foliar and soil applications indicate that both methods are equally effective in enhancing total chlorophyll under drought stress. For chlorophyll *b*, however, the significant increases compared to the control were observed under seed (71% increase), and soil drench (53%) application. Overall, both application methods were not significantly different under selenium treatment (Table 2).

For parameters with no significant three-way or two-way interactions, the main effects of (selenium concentration, application method, and cultivar) were analysed independently. Only the parameters with no significance for the three-way and two-way interactions are reported. Cultivars differed significantly in chlorophyll *b* (*p* ≤ 0.05) at flowering and in EL (*p* ≤ 0.01) at pod filling, indicating genotypic differences between UVE14 and UVE17 for these parameters (Table 1). The method of selenium application had a significant influence on chlorophyll *b* content (*p* ≤ 0.05), EL (*p* ≤ 0.001) and H_2_O_2_ (*p* ≤ 0.01) at the flowering stage. At pod-filling, the selenium application method had an influence on EL (*p* ≤ 0.001) (Table 1). There were no additional effects of selenium treatment alone, apart from those already captured under the significant two-way and three-way interactions. This suggests that the influence of selenium on the measured physio-biochemical parameters studied largely depended on the application method and cultivar, rather than solely on selenium concentration (Table 1).

### 2.2. Photosynthetic Parameters

Table 3 presents the mean photosynthetic efficiency parameters of UVE14 and UVE17 under drought stress with different selenium concentrations and application methods at flowering and pod-filling stages. At flowering, selenium seed priming significantly increased PI_total_ only in the UVE14 cultivar (94% increase). However, selenium soil application improved PI_total_ in both cultivars, with increases of 114% in UVE14 and 62% in UVE17. At pod-filling, selenium soil application continued to enhance PI_total_ in both cultivars, with UVE14 showing a 79% increase and UVE17 a 53% increase. Both seed and foliar selenium treatments were no longer effective at inducing PI_total_ in both cultivars. Foliar treatment had no significant effect on either cultivar at flowering or during pod-filling. Overall, soil application of selenium maintained higher PI_total_ values in both UVE14 and UVE17 at flowering and pod-filling, whereas seed and foliar applications had limited or no significant effects.

Although PI_abs_, Fv/Fm, DIo/RC showed no significance for three-way (Table 1) or selenium × application method interaction (Table 2), there were notable cultivar-specific responses observed. In UVE14 at flowering, foliar selenium application increased PI_abs_ by 88%, whereas no significant changes were detected in UVE17 or under other application methods. Selenium foliar application was the only treatment that significantly enhanced Fv/Fm (4% increase) and reduced DI_0_/RC (31% decrease) in UVE17 under drought stress during the pod-filling stage.

At flowering, seed treatment led to a significant reduction (75%) in stomatal conductance for UVE14. Contrary to pod-filling, stomatal conductance significantly increased (54%) in UVE14. Under foliar application, stomatal conductance was significantly increased for UVE14 (70%) at flowering and pod-filling (35%). Although not significant for UVE17, this treatment increased the stomatal conductance in this cultivar at both reproductive stages. Applying selenium as a soil drench greatly increased stomatal conductance in UVE17 (by 85%) at flowering but decreased it in UVE14. A similar trend was seen at pod-filling, though the increase in UVE17 was not significant.

Table 4 shows the mean photosynthetic pigments’ content in UVE14 and UVE17 under drought stress with different selenium concentrations and application methods at flowering and pod-filling stages. Chlorophyll *a* (chl *a*) and Chlorophyll *b* (chl *b*) are essential for photosynthesis, as they absorb light energy needed for converting CO_2_ and water into sugars. Chlorophyll *a* plays a primary role in driving electron transport, while chl *b* broadens the light absorption range, enhancing overall efficiency. Selenium application as a soil or foliar treatment did not influence chl *a* accumulation in both cultivars. However, seed treatment significantly reduced chl *a* production in UVE14 at the flowering stage, while its effect at pod-filling was insignificant. Selenium application as a seed priming significantly reduced chlorophyll *b* content in UVE14 by 34% at the flowering stage. However, all other selenium treatments did not significantly affect chlorophyll *b* production.

When treated with selenium as a seed primer, UVE14 showed a significant (*p* ≤ 0.05) drop in total chlorophyll at both the flowering and pod-filling stages. In UVE17, chlorophyll levels increased (60%) at flowering but dropped significantly (25% decrease) at pod-filling under this treatment. Selenium foliar application at pod-filling significantly increased total chlorophyll accumulation in both UVE14 (61%) and UVE17 (28%). Similarly, selenium soil application increased total chlorophyll levels in both UVE14 (29%) and UVE17 (46%) at the pod-filling stage.

### 2.3. Relative Water Content

The effects of different selenium application methods on the relative water content (RWC) of drought-stressed vegetable soybean cultivars (UVE14 and UVE17) are given in Figure 1. This physiological parameter helps to assess a plant’s ability to maintain hydration and cope with water loss. At the flowering stage, seed treatment caused a significant decrease in RWC for UVE17, but at pod-filling, it increased RWC for both UVE14 and UVE17. Foliar application of selenium increased RWC in UVE17 (4%) only at the flowering stage. In contrast, at pod-filling, this method significantly reduced RWC in both cultivars. Soil application was not effective for UVE14 cultivar at flowering but was significant for UVE17, which led to a substantial increase in the RWC for cultivar UVE17 (4%). Furthermore, at pod-filling, the application of selenium as a soil drench was only effective for UVE17, which led to a significant increase in RWC (11%).

### 2.4. Hydrogen Peroxide

Figure 2 represents the hydrogen peroxide (H_2_O_2_) content in two drought-stressed vegetable soybean cultivars grown under the different selenium application methods. H_2_O_2_ is one of the reactive oxygen species (ROS) produced under drought stress. Depending on the level of accumulation, H_2_O_2_ could be destructive to plant cells or be beneficial, where it can act as a signalling molecule for stimulating the defence mechanisms. Seed treatment significantly reduced H_2_O_2_ accumulation in UVE14. In contrast, soil selenium applications increased H_2_O_2_ levels in UVE14 at pod-filling (49% increase).

### 2.5. Antioxidative Responses

Antioxidative mechanisms such as carotenoids, ascorbate peroxidase (APX), Guaiacol peroxidase (GPX), and superoxide dismutase (SOD) play a crucial role in maintaining ROS balance within plant cells. Figure 3A represents the carotenoid content of drought-stressed vegetable soybean cultivars grown under the different selenium application methods. During the flowering stage, there was a significant increase in carotenoid content (32%) in cultivar UVE17, while a notable decrease (36%) was observed in UVE14 under the seed application method. Foliar application significantly increased carotenoid content in UVE14 (26%) but decreased it in UVE17. Notably, at the pod-filling stage, soil treatment increased carotenoid content significantly for both cultivars, UVE14 (45%) and UVE17 (54%).

Ascorbate peroxidase activity of vegetable soybean grown under drought stress for the different selenium application methods is given in Figure 3B. The APX activity was significantly reduced in UVE14 under the selenium seed priming method (33%) at pod-filling. Foliar application had no effect on increasing this enzyme activity, while soil application effectively enhanced APX activity at pod-filling in UVE14 (223% increase).

Figure 3C represents the GPX activity of drought-stressed vegetable soybean under the different selenium application methods. Soil drenching with selenium significantly increased GPX activity in UVE14 (33%) at flowering but reduced it in UVE17 at pod-filling. In UVE17, seed priming lowered GPX activity at both stages, while foliar application reduced this enzyme’s activity at flowering in UVE14.

Figure 3D represents the SOD activity of drought-stressed vegetable soybean cultivars grown under different selenium application methods. At the flowering stage, selenium seed treatment significantly increased SOD activity in UVE17 (27%) while reducing it in UVE14. Furthermore, at pod-filling, this treatment increased this activity in UVE14 (222%). Application of selenium as a foliar spray induced a significant increase in SOD activity in UVE17 (98%) at pod-filling stage. Selenium soil treatment significantly increased SOD activity in UVE14 (24%) at flowering, while it reduced in UVE17 at pod-filling.

### 2.6. Growth and Yield Traits

Table 5 presents the main effects of selenium (Se), application method (M), and cultivar (C), as well as their two-way (Se *×* M) and three-way (Se *×* M *×* C) interactions, on the growth and yield parameters of two vegetable soybean cultivars (UVE14 and UVE17) at the reproductive stage eight under drought stress (ANOVA). A significant three-way interaction (Se *×* M *×* C) was observed for plant height (PH) (*p* ≤ 0.05) and the number of seeds per plant (SPP) (*p* ≤ 0.001). These significant interactions indicate that the effect of selenium application on these parameters varied between cultivars and depended on the method of application (Table 5).

In contrast, number of branches per plant (BPP) and number of pods per plant (PPP) were not significant at the three-way interaction level. However, a significant selenium × application method interaction (*p* ≤ 0.001) was observed for both parameters, indicating that their responses were primarily influenced by the combined effects of selenium concentration and method of application (Table 5). To gain deeper insight into these interactions, the analysis was further partitioned to evaluate the main effects of selenium concentration and application method within each treatment combination (Table 6). Compared to the untreated, drought-stressed control, selenium seed application was not significant for both parameters. In contrast, foliar selenium application significantly increased the number of branches per plant (BPP) by 24%, while the soil drenching method led to an 18% increase in the number of pods per plant (PPP) relative to the control. These findings show that the method of selenium application substantially influenced both BPP and PPP under drought conditions.

Table 7 depicts the influence of different selenium application methods on the growth and yield attributes of two vegetable soybean cultivars (UVE14 and UVE17) subjected to drought stress. A foliar spray led to a significant increase (60%) in the number of branches per plant (BPP) for UVE17. In contrast, selenium application as a seed primer or soil drench did not affect this trait in either cultivar. Selenium application significantly reduced plant height (PH), with soil application affecting UVE17 and foliar application affecting UVE14. However, seed priming method increased PH in drought-stressed UVE14.

Compared to the control group in UVE17, selenium foliar spray notably elevated the number of pods per plant (PPP) by a significant 33% during drought stress. Similarly, selenium application as a soil drench increased PPP in UVE17 by a significant 21% compared to the control. Conversely, selenium seed application treatment did not induce noteworthy improvements in PPP under drought stress conditions. A comparative analysis of the three selenium application methods revealed that the most substantial increase occurred under soil drench treatment in drought-stressed UVE17.

In comparison to the control group, selenium seed application did not show a significant improvement in the number of seeds per plant (SPP) in UVE14, but it significantly increased this parameter by 29% in UVE17 under drought stress. Selenium foliar application did not affect the SPP in either cultivar under drought stress. The soil drench method showed the most significant increase in SPP, resulting in a 48% improvement in drought-stressed UVE17. In contrast to UVE14, this application method resulted in a reduction in this trait under drought stress.

## 3. Discussion

Drought stress, exacerbated by climate change, is one of the biggest challenges to crop production. In legumes like vegetable soybean, stable yields under drought conditions depend on the ability of plants to maintain optimal physiological and biochemical function [13,28,29,30]. Many strategies have been explored to improve drought survival in legumes [29]. However, little research has focused on the role of selenium, despite its great potential to improve plant tolerance to drought stress [31]. Moloi and Khoza [18] demonstrated that foliar spraying of selenium on vegetable soybean leaves during the vegetative stage enhanced drought tolerance by increasing APX activity. However, the efficacy of this application method appeared to decline over time, as only APX activity was elevated at the pod-filling stage without any corresponding improvement in yield. This highlights the need to investigate alternative selenium application methods, such as seed priming and soil treatments, which have shown potential to improve drought tolerance in plants [21,32]. This study investigates the effects of different selenium application methods (seed priming, soil drenching, and foliar spraying) on the physio-biochemical responses and yield traits of two vegetable soybean cultivars with varying drought tolerance levels. The effectiveness of these selenium application methods is measured over time (at flowering and pod-filling stages) because different developmental stages in plants experience different physiological and biochemical responses to drought stress [33]. Since drought stress, especially during critical growth periods, can cause irreversible yield losses [34], optimising selenium application methods is essential for finding alternative solutions to genetic engineering or conventional breeding for enhancing drought resilience and maximising yield in vegetable soybean.

Selenium soil drench application improved photosynthetic efficiency under drought conditions, as evidenced by increased total chlorophyll content and photosystem performance (PItotal) in both vegetable soybean cultivars (UVE14 and UVE17) across growth stages. This suggests that selenium enhances the photosynthetic apparatus of and supports physiological stability under drought stress. However, the response was growth stage-dependent, with a notable increase in total chlorophyll accumulation observed only at the pod-filling stage, indicating that selenium effectiveness is influenced by developmental stage. Similarly, the application of selenium as a soil supplement increased the chlorophyll content of drought-stressed lentils [35], indicating that selenium can regulate physiological processes [36]. Under drought stress, plants respond by inducing stomatal closure to reduce transpiration. While this is a survival mechanism, stomatal closure reduces CO_2_ fixation, leading to reduced plant growth [37,38]. Selenium soil application method effectively increased stomatal conductance in drought-stressed UVE17 at both growth stages. These imply that selenium soil application enhanced the light harvesting and light-dependent electron transport irrespective of the cultivar under drought conditions. Under drought stress, plants typically close their stomata to reduce water loss. However, this treatment increased stomatal conductance in UVE17 at pod-filling, suggesting higher CO_2_ uptake and enhanced metabolite production that support crop growth and yield [39]. Interestingly, soil-applied selenium decreased stomatal conductance in UVE14, indicating that the treatment’s ability to improve stomatal behaviour and drought tolerance is more pronounced in cultivars that are inherently more susceptible to water stress. The increased relative water content (RWC) observed in drought-stressed UVE17, at the pod-filling stage, suggests improved water retention, which supports survival and maintains optimal physiological processes [40]. The observed increase in RWC in UVE17 under soil-applied selenium may be related to the reduced plant height, suggesting that shorter stature can help conserve water and support physiological stability under drought stress. Additionally, the ability of UVE17 to sustain high RWC despite elevated stomatal conductance with selenium soil drench treatment may be attributed to osmotic adjustment, which facilitates water retention under stress conditions [41]. Nano-selenium application had the same effect on other crops’ RWC as in this study. In strawberry and pak choi, nano-selenium (and Se/SiO_2_ nanocomposites) under drought stress significantly increased RWC, improved membrane stability, and enhanced antioxidant activity, supporting better water retention and physiological function during critical growth stages [23,25]. Studies also show that application of selenium on drought-stressed plants increases tolerance to drought by preventing oxidative damage through the activation of their antioxidative system [15,35]. Selenium application in the soil further increased hydrogen peroxide (H_2_O_2_) accumulation in the drought-stressed UVE14 cultivar. This increase may be linked to the observed reduction in stomatal conductance under selenium treatment, as stomatal closure can lead to the accumulation of reactive oxygen species (ROS), including H_2_O_2_ [42]. Although H_2_O_2_ levels increased with selenium soil drench application in UVE14, the mean was lower compared to that of seed and foliar applications, indicating that soil drenching with selenium only induced moderate H_2_O_2_ levels, suggesting limited ROS damage from drought stress. Also, a lower H_2_O_2_ level is crucial for drought tolerance, as it serves as a signal molecule for activating drought tolerance in vegetable soybean [30]. To keep ROS levels low under drought stress, plants need functional antioxidative responses to scavenge ROS, reducing the risk of oxidative stress that can damage vital biological molecules like lipids and proteins essential for cellular functions. In UVE14, selenium soil drench application under drought conditions enhanced most antioxidative responses, including carotenoids, ascorbate peroxidase, guaiacol peroxidase, and superoxide dismutase. Previous studies on vegetable soybean have shown that this cultivar enhances drought tolerance by activating ROS scavenging mechanisms [13,30]. Compared to the control, the observed increases in various antioxidative systems indicate that while the antioxidative response is inherently activated, selenium soil application further enhances ROS scavenging activity, strengthening UVE14’s ability to mitigate oxidative stress. In contrast, selenium soil application only increased carotenoid content in UVE17 under drought conditions, showing that enzymatic antioxidative mechanisms are not part of the drought tolerance responses in this susceptible cultivar. In addition, Zeeshan et al. [22] also had similar findings using nano-selenium, they found that nano-selenium application improved oxidative stress management in drought-stressed soybean during the reproductive stage. Since yield is a critical factor in agriculture and food security, it is essential to determine whether the physiological and biochemical tolerance improvements observed under selenium soil application translate into enhanced growth and yield. Selenium supplementation through a soil treatment was beneficial for drought-stressed lentils, resulting in significant improvements in the pod number and seed yield per plant [43]. For the yield parameters, selenium soil application could significantly increase the total seed number per plant (SPP) and total pods per plant (PPP) only in UVE17 under drought stress. Notably, in drought-stressed UVE17 at pod-filling, the increase in PPP under this selenium application method correlates positively with total chlorophyll content (r = 0.81) and RWC (0.85) (Table S1), confirming that improved physiological traits could contribute to the observed yield benefits under drought conditions [8]. In contrast, for drought-stressed UVE14, selenium soil application could only enhance one yield parameter, SPP. Furthermore, correlations show that an increased SPP in drought-stressed UVE14 under selenium soil treatment could be explained by a corresponding increase in the total chlorophyll (r = 0.89), stomatal conductance (r = 0.97) and SOD (r = 0.86) (Table S1), suggesting that soil treatment was very efficient in improving tolerance for both cultivars.

Like selenium soil-drench, foliar application also increased total chlorophyll content in both UVE14 and UVE17 at the pod-filling stage. However, its effects on other traits depended on the cultivar and growth stage. In UVE17, selenium foliar spraying improved photosynthesis efficiency by increasing the maximum quantum efficiency of photosystem (PS) II (Fv/Fm) and lowering non-photochemical quenching, implying more efficient photochemical reactions. The reduced RWC in this cultivar, despite selenium foliar treatment under drought conditions, is linked to partially opened stomata, which suggests increased water loss with no osmotic adjustment. The selenium application consistently lowered H_2_O_2_ accumulation, with the strongest reductions observed for soil drenching, followed by seed priming and foliar spraying. The drought-susceptible cultivar (UVE17) exhibited slightly greater percentage decreases than the drought-tolerant cultivar (UVE14), indicating a potentially higher oxidative stress mitigation benefit from Se supplementation in this genotype (Figure 3). These results correspond with the observation of Zeeshan et al. [22]. They stated that foliar application of SeNPs during the reproductive stage in soybean significantly increases above-ground biomass, relative water content, and photosynthetic pigments under drought. SeNPs also boost antioxidant enzyme activity and osmolyte accumulation, reducing oxidative damage and improving cellular integrity. SeNPs improve stomatal function, mesophyll cell structure, and reduce reactive oxygen species (ROS) and membrane lipid peroxidation, leading to better drought resilience. Similarly, Sattar et al. [44] reported an increased stomatal conductance in drought-stressed tomatoes under selenium foliar application. Of the antioxidative mechanisms studied, selenium foliar application increased UVE17’s potential of converting the superoxide anions to H_2_O_2_ through increased superoxide dismutase (SOD) activity, which, however, did not translate into improved yield parameters (no significant positive correlations with the yield parameters, Table S2). The increased number of branches per plant (BPP) and pods per plant (PPP) in drought-stressed UVE17 under this selenium application method cannot be explained by the observed physio-biochemical responses (no correlations), suggesting the involvement of alternative mechanisms. In contrast to the previous study by Moloi and Khoza [18], which showed no increase in the yield parameters when selenium was applied at V1 stage, foliar spraying of selenium slightly later (V2 stage) improves yield performance. This suggests that the efficacy of selenium when applied foliarly reduces with time. In support, Xia et al. [43] suggested that the differences in yield parameters between soil and foliar in wheat under drought stress were influenced by the time of selenium application and the organ of the plant where selenium was applied (soil or foliar).

Selenium is known to enhance antioxidant defence, reduce oxidative stress, and help maintain chlorophyll and PSII efficiency under drought in various crops, but its impact is often context- and stage-dependent [45,46,47,48]. In drought-stressed UVE14, this application method also enhanced the light-harvesting capacity (total chlorophyll) and PSII (PI_abs_) efficiency, along with increased stomatal conductance and better water retention. Improved leaf water status was also reported under combined heat and drought stress in maize when selenium was applied foliarly [49]. Similarly, Rady et al. [20] showed that foliar application of selenium increased RWC in drought-stressed wheat. This evidence suggests that while selenium application can mitigate drought stress by enhancing water retention in plant tissues [50], its effectiveness varies by cultivar. Unlike soil application, foliar application in drought-stressed UVE14 increased H_2_O_2_ levels and carotenoid content. However, selenium foliar spraying decreased plant height (PH) but did not enhance any yield traits in this cultivar under drought stress. Furthermore, the findings correspond to the results of a study performed on quinoa under drought stress [51], where it was found that while selenium foliar application improved physiological traits such as chlorophyll content and antioxidant activity, it did not increase the yield components. Xia et al. [43] also illustrated that selenium application through foliar did not yield more grain in two purple wheat cultivars under drought stress.

Pre-treatment of vegetable soybean seeds with selenium had different effects on the physio-biochemical responses of drought-stressed vegetable soybean cultivars. In drought-stressed UVE14, selenium treatment enhanced the overall efficiency of PSII and PSI and increased the likelihood of CO_2_ fixation, as indicated by higher stomatal conductance at the pod-filling stage. Notably, RWC at this stage improved despite increased stomatal opening, suggesting that this selenium application method may have activated additional drought tolerance mechanisms that prevent desiccation, thereby enhancing RWC, consistent with the findings of Sita et al. [35]. Similarly, the selenium-mediated increase in maize yield was related to the maintenance of turgor and increased water retention, suggesting less cell membrane damage, which helps plants produce more biomass under water stress conditions [36]. However, further investigations are needed to clarify the mechanisms used by drought-stressed UVE14 to maintain high RWC in drought-stressed UVE14 under the selenium seed priming method. Although carotenoid content declined in this cultivar under drought stress and selenium treatment at flowering, other ROS scavenging mechanisms, particularly increased GPX activity at flowering and SOD activity at pod-filling, effectively neutralised H_2_O_2_, as evidenced by its reduced levels. Similarly, Ibrahim [52] reported that selenium pre-treatment through seed soaking reduced H_2_O_2_ accumulation in wheat, suggesting a crucial role for selenium in ROS inhibition during drought stress [21]. While selenium seed-soaking effectively enhanced the physio-biochemical responses in drought-stressed UVE14, it did not significantly improve the yield traits, as no strong positive correlations were observed with these parameters (Table S3). Pre-treatment of UVE17 seeds with selenium enhanced the light-harvesting capacity by increasing chlorophyll content at flowering and strengthened antioxidative responses, as indicated by higher carotenoid levels and SOD activity under drought stress. Selenium also mediated the increased carotenoid content of cold-stressed wheat seedlings [53]. This selenium application method was effective in improving drought tolerance because not only did it increase the above physio-biochemical responses, but it also increased the SPP in a drought-susceptible UVE17. The strong positive correlations between these parameters at flowering and the SPP in drought-stressed UVE17 suggest that seed soaking with selenium induces distinct drought tolerance mechanisms across cultivars (Table S3).

## 4. Materials and Methods

This section outlines the physiological and biochemical measurements conducted to achieve the study’s objectives. This included chlorophyll *a* fluorescence parameters (to assess the efficiency of the light-dependent electron transport in photosynthesis), photosynthetic pigments (important for light harvesting and photoprotection of the photosynthesis apparatus [54]), and stomatal conductance (regulates CO_2_ entry for the light-independent reactions).

Additional analyses included relative water content (describes the amount of water in a leaf at sampling time, which is an important indicator of stress in plants [55]) hydrogen peroxide content (a ROS produced under drought stress), electrolyte leakage (indicative of damage induced by overproduction of ROS), and antioxidative enzyme activities (involved in ROS scavenging to maintain cellular balance).

### 4.1. Plant Material, Growth Conditions, Selenium Application, and Drought Conditions

To ensure that the observed responses are not specific to a particular cultivar, two vegetable soybean varieties, namely the drought-susceptible (UVE17) and drought-tolerant (UVE14), as classified by van der Merwe et al. [6], were used. Vegetable soybean seeds were provided by the South African Edamame Development Program as the breeder of the cultivars. This approach aimed to confirm if the measured responses are more likely to be general phenomena or specific to a particular cultivar. The seeds were germinated under controlled conditions (25 °C day and 18 °C night) in polystyrene seedling trays filled with Hygromix seedling mix (Hygrotech (Pty) Ltd., Pretoria, South Africa), with the chemical composition of peat moss > 40%, vermiculite > 20%, and polystyrene. At the unifoliate leaf (UV) stage, the seedlings were transplanted into potting bags with the following dimensions: 350 mm length, 150 mm width, filled with dried loamy sandy soil (10 kg), with one seedling per pot. The soil was watered to 100% water holding capacity (WHC) with 1.6 L H_2_O, bringing the soil weight to 11.6 kg (Optika balance, Optika S.r.l., Ponteranica, province of Bergamo, Italy). The trial design was a completely randomised block design with three replications.

A Hydrosense II meter, attached to a 12 cm sensor probe (CS659; Campbell Scientific, Stellenbosch, South Africa) was used to determine the amount of water needed to maintain the soil at 100% WHC. The volumetric water content (VWC) of the soil at this level was used to calculate the amount of water required daily to avoid water stress. To avoid nutrient deficiencies, plants were fertilised with full-strength (200 mL) Hygrotech nutrient solution, consisting of essential macro- and micronutrients, every two weeks until the end of the pod-filling stage.

For seed treatment, six seeds per cultivar were submerged in 200 mL sodium selenate (Na_2_SeO_4_, 50 mg/L) solution, followed by sowing in the seedling trays as mentioned above (the control was submerged in double-distilled water). For the soil drench method, 200 mL of selenium (50 mg/L) was added to the soil (the control was drenched with 200 mL of double distilled water) at the V2 stage. Similarly, at V2 stage, foliar selenium (50 mg/L, prepared in 200 mL) was sprayed until leaf droplets formed without dripping; controls received double-distilled water. The selection of a 50 mg/L concentration was guided by a prior study [18], which identified the dose as within the optimal range for inducing the specific physio-chemical parameters in vegetable soybean under drought stress. The soil had a pH of 8.64, a soil redox of 217 M/V, and an electrical conductivity (EC) of 41 μS. The water holding capacity (WHC) was 1.6 L per 10 kg of soil (100% WHC). The total selenium content in the untreated soil was measured at 487 mg/kg, and macronutrient and micronutrient levels included nitrogen (0.048%), phosphorus (10 mg/kg), potassium (261.9 mg/kg), iron (9.8 mg/kg), calcium (408.83 mg/kg), and copper (0.043 mg/kg).

The method of measuring soil pH was according to Black [56]. A soil sample of 5 g was added to a 100 mL conical flask filled with 50 mL of distilled water. The pH was measured using a Hanna HI98121 pH/ORP/Temperature Tester (three calibrated at 20 °C).

The redox potential of the soil was determined according to Barnard [57]. A Hanna HI98121 pH/ORP/Temperature Tester (three-point-calibrated at 20 °C) was inserted into distilled water (25 mL) with 12.5 g of soil in a conical flask. The solution was shaken for 30 min, and the reading was taken.

The soil electrical conductivity of a saturated paste was determined according to Barnard [57]. Deionised water was added to a beaker containing a 250 g sample of soil until it was completely saturated. The soil was evaluated for the characteristics of a saturated paste, i.e., no surplus water on the surface of the soil. The saturated paste was let to stand for at least an hour before being filtered using Whatman No. 40 filter paper and a Buchman filter. A Metrohm Touch Control Swiss mode was used to test the temperature and conductivity of the filtrate.

Plants were continuously watered to 100% WHC until the initiation of drought stress. Drought stress was induced by withholding irrigation until 30% WHC at the third trifoliate leaf stage (V4). Irrigation was then maintained at either 30% or 100% WHC until the completion of the plant life cycle. Physiological and biochemical measurements were performed at the flowering and pod-filling stages, as these are the most sensitive to drought [30]. Young but fully expanded trifoliate leaves were used for both non-destructive (chlorophyll fluorescence and stomatal conductance) and destructive (chloroplast pigments, hydrogen peroxide, and antioxidative enzymes) measurements, as they are actively growing. Leaf sampling and non-destructive measurements were conducted between 09:00 a.m. and noon.

### 4.2. The Physiological Measurements

A pocket photosynthesis efficiency analyser (PEA, Hansatech Instrument Ltd., Pentney, Norfolk, UK) was used to measure the effects of different selenium application methods on the photosynthetic efficiency of drought-stressed vegetable soybean at the flowering and pod-filling stages. To ensure repeatability, chlorophyll *a* fluorescence data were collected three times for each young, fully expanded trifoliate leaf. Sample leaves were dark-adapted for 30 min using lightweight leaf clips with closed shutter plates. The clips were placed to the middle part of leaves, avoiding the midrib and leaf edges. Measurements included the maximum quantum efficiency of photosystem II (Fv/Fm), total performance index (PI_total_), performance index for absorbance (PI_abs_), and energy dissipation as heat (DI_0_/RC).

The method described by Su et al. [58] was used to determine the concentrations of chlorophyll a (chl-*a*), chlorophyll b (chl-*b*), and carotenoid. Frozen leaf samples (0.1 g) were extracted with ice-cold 80% (*v*/*v*) acetone (5 mL) using a pestle and mortar on ice. The extract was centrifuged at 11,000× *g* for 10 min, and the absorbance of the supernatant was measured at 663, 645, and 480 nm. The total chlorophyll, chl-*a*, chl-*b*, and carotenoid contents were calculated according to Pareek et al. [59].

A leaf porometer SC-1 (Li-Cor. ADC BioScientific Ltd., Hoddesdon, UK), which quantifies the humidity gradient between the chamber and the leaf surface surroundings, was used to measure stomatal conductance on young, fully expanded trifoliate leaf.

Relative water content (RWC) was determined according to the method described by González and González-Vilar [60]. Leaves were placed in airtight 50 mL Falcon tubes to prevent moisture loss. Fresh weight (FW) was recorded immediately in the laboratory. After adding 10 mL of double-distilled water, the tubes were incubated at 4 °C for 24 h and the turgid weight (TW) was recorded. After oven-drying at 72 °C for 72 h, dry weight (DW) was recorded. The RWC (%) was calculated using the formula; RWC (%) = (FW − DW)/(TW − DW) × 100.

### 4.3. The Biochemical Measurements

A modified method described by Velikova et al. [61] was used for the H_2_O_2_ assay. Trichloroacetic acid (TCA, 0.1% *w*/*v*, 1 mL) was added to 0.1 g of frozen leaf tissue, homogenised on ice, and centrifuged at 12,000× *g* for 15 min at 4 °C. To the supernatant (0.25 mL), 0.25 mL of 10 mM potassium phosphate buffer (pH 7.0) and 0.5 mL 1 M potassium iodide were added. For the blank, 0.25 mL of 0.1% (*w*/*v*) TCA was used in place of the supernatant. Absorbance was measured at 390 nm (Cary 100 Bio, Varian, Australia) after the reaction developed for 1 h in the dark at room temperature.

#### 4.3.1. Enzyme Extract Preparation

Enzyme extracts were prepared according to Pukacka and Ratajczak [62]. Leaves were ground in liquid nitrogen to fine powder using a mortar and pestle. Leaf powder (0.1 g) for each treatment was homogenised on ice in 1 mL of 50 mM potassium phosphate buffer (pH 7.0) containing 1 mM EDTA, 2% (*w*/*v*) PVPP, 0.1% (*v*/*v*) Triton X-100. and 1 mM ascorbate (to oxidise the intracellular H_2_O_2_). The homogenate was centrifuged at 15,000× *g* for 20 min at 4 °C. The supernatant was used as the enzyme extract and assayed in triplicate for all enzyme analyses.

#### 4.3.2. Antioxidative Enzyme Assays and Protein Content

The ascorbate peroxidase (APX) assay was performed according to the modified method of Mishra et al. [63]. The reaction mixture (1 mL) consisted of 500 μL of 50 mM potassium phosphate buffer (pH 7.0), 200 μL of 0.1 mM H_2_O_2_, 150 μL of 0.5 mM sodium ascorbate, and 150 μL of enzyme extract. Ascorbate oxidation was measured at 290 nm (Cary 100 Bio, Varian, Australia) for 3 min at 30 °C against a blank in which the enzyme extract was replaced with phosphate buffer.

Guaiacol peroxidase (GPX) activity was determined following the method of Zieslin and Ben-Zaken [64]. The reaction mixture (1 mL) consisted of 500 µL of 80 mM potassium phosphate buffer (pH 5.5), 50 µL of 0.2 M H_2_O_2_, 100 µL of 50 mM guaiacol, 340 µL distilled water, and 10 µL of enzyme extract. The absorbance was monitored at 470 nm (Cary 100 Bio, Varian, Australia) over 3 min at 30 °C.

Superoxide dismutase (SOD) activity was determined using a modified method of Hwang et al. [65]. Enzyme extract (15 μL) was mixed with 1.28 mL of 100 mM potassium phosphate buffer (pH 7.8), 40 μL of 55 mM methionine, 150 μL of 0.75 mM nitroblue tetrazolium (NBT), and 30 μL of 0.1 mM riboflavin. The cuvettes containing reaction mixtures were irradiated under two light bulbs (1000 kW) placed side by side (3 cm apart) and a high of 28 cm for 30 min. The control, prepared in the same way, contained all reactants except the enzyme. A non-irradiated reference was kept in the dark for the duration of the experiment. One unit of SOD activity was defined as the amount of enzyme required to inhibit 50% of NBT photoreduction.

Protein content was quantified using the Bradford method [66], with gamma globulin serving as the standard (0.5 mg/ mL), in a 96-well microplate (Greiner Bio-One, Kremsmunster, Austria). The absorbance was measured at 595 nm using a microplate reader (Anthos Labtech Inc., GmbH, Salzburg, Austria).

Three replicates per treatment and three technical replicates per measured parameter.

### 4.4. Yield Traits

The following growth and yield traits were measured at R8 stage: plant height (PH), number of branches per plant (BPP), number of pods per plant (PPP) and number of seeds per plant (SPP).

### 4.5. Statistical Analysis

For the statistical analysis, data were subjected to a three-way analysis of variance (ANOVA) using Statistica version 7 (TIBCO Software Inc., Palo Alto, CA, USA) to test the effects of selenium treatment (Se), application method (seed, foliar, and soil), and cultivar (UVE14 and UVE17), as well as their interactions, on physiological and biochemical parameters. When significant main or interaction effects were detected, Fisher’s LSD was used for post hoc comparisons to determine differences between means at *p* ≤ 0.05.

For parameters showing significant three-way interactions (Se × Method × Cultivar), simple two-way interactions were further analysed within each cultivar to identify specific treatment differences. Means followed by different lowercase letters indicate significant differences (*p* ≤ 0.05) among treatment combinations within each cultivar and growth stage, whereas shared letters indicate no significant difference.

Statistical analyses were performed separately for each growth stage (flowering and pod-filling) to assess stage-specific responses. The relationship between yield and physio-biochemical parameters was analysed using correlation matrices, providing regression coefficients and *p*-values at a 95% confidence level.

## 5. Conclusions

The effectiveness of selenium in enhancing physiological, biochemical, and yield responses of vegetable soybean under drought stress is both cultivar and application-specific. Soil-applied selenium markedly improved drought tolerance in UVE17 by enhancing photosynthetic efficiency, increasing stomatal conductance, reducing plant height while maintaining high RWC, and regulating ROS homeostasis, which translated into higher seed and pod numbers (SPP and PPP). In UVE14, soil application primarily enhanced physiological and biochemical traits, resulting in improved SPP only. Foliar selenium application improved selected physiological and biochemical responses in a cultivar-dependent manner, increasing yield parameters (number of branches per plant (BPP) and PPP) in UVE17 but not in UVE14. In contrast, seed priming decreased photosynthetic efficiency in UVE14, while enhancing certain physiological and biochemical responses, but without improving yield. In UVE17, it increased the seed number per plant (SPP). These findings emphasise that selenium’s impact depends on both cultivar and application method, with more pronounced benefits in drought-susceptible cultivars. Soil application provides a more practical approach to sustaining productivity under water-limited conditions. Future studies should elucidate the mechanisms underlying selenium-mediated drought resilience, including maintenance of high RWC despite open stomata, and investigate the effects of application timing on physiological, biochemical, and yield outcomes.

## Figures and Tables

**Table 1:** Analysis of variance (ANOVA) showing the main effects and interactions of cultivar, selenium concentration, and application method on physiological and biochemical parameters of edamame at different growth stages.

		Flowering							Pod Filling	
	Selenium (Se)	Method (M)	Cultivar (C)	Se *×* M	Se *×* M *×* C	Selenium (Se)	Method (M)	Cultivar (C)	Se *×* M	Se *×* M *×* C
Fv/Fm	0.0005	0.000134	0.00005	0.00006	0.00015	0.0003	0.000	0.000107	0.00033	0.00
PI_total_	20.03 ***	6.036913 **	1.35 **	5.0677 *	0.6862 **	8.85	4.19	2.185855	5.9289	0.57
PI_abs_	16.02	2.986683	4.126992	1.278	1.49	0.001	0.005	4.628952	6.611	3.08
DIo/RC	0.00184	0.000557	0.000868	0.000082	0.00110	0.001	0.005	0.003731	0.003263	0.006
ABS/RC	0.212	0.060826	0.162073	0.05080	0.10410	0.021	0.07	0.025531	0.03602	0.029
ETo/RC	0.0037	0.009919	0.001746	0.00215	0.00054	0.00001	0.001	0.00046	0.002469	0.001
RWC	19.50	33.92117	108.92 ***	44.384 *	57.601 **	1.292	44.69 ***	2.087	300.784 ***	80.91 ***
Chl *a*	0.028	0.023812	0.119039	0.00603	0.06652	0.084	0.028	0.0013	0.19462	0.271
Chl *b*	0.01806	0.040 *	0.051 *	0.011691	0.023	1.80	10.10 **	82.62 ***	38.522 ***	4.271
Tot chl	0.22 *	0.175 *	0.101147	0.10308	0.389 ***	0.37 ***	0.149 *	0.915 ***	0.5659 ***	0.024
Car	0.000066	0.000022	0.0002 ***	75,126.387 **	39.872 ***	30,785 **	99,123 ***	68,735 ***	16,723.698 *	16,567 *
g_s_	1839.33	33,961.5	286,461 ***	0.000025	143,152 ***	0.0003 ***	0.0004 ***	0.0006 ***	0.0003 ***	16,567 *
APX	0.306	0.636	0.232	0.60945	0.533	3.047 *	4.90 *	12.46 **	8.55177 ***	4.025 **
GPX	117.30 ***	44.63 ***	1.262	2.64	109.07 ***	3.54	97.51 ***	80.72 ***	19.2100 *	12.91 ***
SOD	506	475	16,703 ***	623.7	1479 **	446 **	1529 ***	1993 ***	1833.17 ***	1198 ***
EL	0.0039	1.7780 ***	0.330	0.0485	0.02	0.028	0.82 ***	0.77 **	0.0375	0.0088
H_2_O_2_	4.524	5.491 **	0.709103	0.8538	2.88	1.42	10.93 ***	0.47071	0.8538	6.12 ***

* *p* ≤ 0.05, ** *p* ≤ 0.01, *** *p* ≤ 0.001. Asterix indicates the level of significance. Fv/Fm: ratio of variable fluorescence to maximum fluorescence, PIabs: performance index absorbance, PItotal: total performance index, DIo/RC: flux of energy as dissipated (heat) per reaction centre (RC), ETo/RC: the flux of electrons transferred from quinone A (QA) to plastoquinone (PQ) per RC, ABS/RC: energy flux for light absorption per RC, Chl *a:* chlorophyll a, Chl *b:* chlorophyll b, Tot Chl: total chlorophyll, g_s_: stomatal conductance, APX: ascorbate peroxidase, GPX: guaiacol peroxidase, SOD: superoxide dismutase, EL: electrolyte leakage, H_2_O_2_: hydrogen peroxidase. Values represent the mean squares.

**Table 2:** The two-way interaction between selenium concentration and application method on the mean total chlorophyll and chlorophyll *b* of drought-stressed edamame at the pod-filling stage.

Selenium Concentration	Method	Total Chl	Chl *b*
0 mg/L	Seed	1.4104 ^b^	0.406 ^a^
50 mg/L		1.111 ^a^	0.695 ^b^
0 mg/L	Foliar	1.188 ^a^	0.448 ^a^
50 mg/L		1.645 ^c^	0.496 ^a^
0 mg/L	Soil	1.25 ^ab^	0.434 ^a^
50 mg/L		1.701 ^c^	0.666 ^b^

The values represent the mean ± SE (*n* = 3). Fisher’s LSD post hoc test was used to determine the significance of differences between the averages (*p* < 0.05). Treatments with different letters (within the columns) are significantly different. If treatments share one or more letters, they are not statistically different. Chl *b*: chlorophyll *b*, Tot Chl: total chlorophyll.

**Table 3:** Mean photosynthetic efficiency parameters of UVE14 and UVE17 under drought stress with different selenium concentrations and application methods at flowering and pod-filling stages.

		PI_total_ (a.u)		PI_abs_ (a.u)		Fv/Fm (a.u)		DI_o_/RC (a.u)		SC (mmol m^−2^ s^−1^)	
Method		Flow	Pod	Flow	Pod	Flow	Pod	Flow	Pod	Flow	Pod
	UVE14 D	2.57 ^a^	4.39 ^abc^	3.38 ^a^	6.66 ^ab^	0.82 ^a^	0.83 ^b^	0.26 ^a^	0.18 ^a^	515.7 ^cde^	118.8 ^a^
	UVE14 D+Se	***4.98 ^cd^***	4.01 ^abc^	6.17 ^ab^	5.54 ^a^	0.83 ^a^	0.83 ^b^	0.23 ^a^	0.20 ^a^	***125.8 ^a^***	***253.4 ^bcde^***
Seed	UVE17 D	3.89 ^abc^	3.93 ^abc^	4.75 ^ab^	7.65 ^abc^	0.81 ^a^	0.83 ^b^	0.23 ^a^	0.18 ^a^	413.8 ^cd^	164.1 ^ab^
	UVE17 D+Se	3.05 ^ab^	3.68 ^ab^	5.11 ^ab^	5.32 ^a^	0.82 ^a^	0.83 ^b^	0.24 ^a^	0.20 ^ab^	566.8 ^de^	235.7 ^bcd^
	UVE14 D	3.28 ^abc^	4.60 ^abc^	4.32 ^a^	7.35 ^abc^	0.81 ^a^	0.83 ^b^	0.27 ^a^	0.19 ^a^	142.5 ^a^	224.0 ^abcd^
	UVE14 D+Se	4.79 ^bcd^	4.64 ^abc^	***8.13 ^b^***	6.83 ^ab^	0.83 ^a^	0.83 ^b^	0.20 ^a^	0.20 ^a^	***352.1 ^bc^***	***346.9 ^ef^***
Foliar	UVE17 D	3.75 ^abc^	4.46 ^abc^	4.96 ^ab^	6.7 ^ab^	0.83 ^a^	0.80 ^a^	0.24 ^a^	0.29 ^b^	541.2 ^de^	425.5 ^fg^
	UVE17 D+Se	3.63 ^abc^	6.03 ^cd^	4.82 ^ab^	8.29 ^bc^	0.82 ^a^	***0.83 ^b^***	0.27 ^a^	***0.20 ^a^***	667.2 ^e^	502.6 ^g^
	UVE14 D	3.32 ^abc^	3.21 ^a^	5.66 ^ab^	7.53 ^abc^	0.83 ^a^	0.83 ^b^	0.24 ^a^	0.18 ^a^	556.2 ^de^	312.2 ^de^
	UVE14 D+Se	***7.10 ^e^***	***5.74 ^bcd^***	6.83 ^ab^	7.38 ^abc^	0.83 ^a^	0.84 ^b^	0.21 ^a^	0.17 ^a^	***84.9 ^a^***	***183.2 ^abc^***
Soil	UVE17 D	3.59 ^abc^	4.51 ^abc^	5.37 ^ab^	7.66 ^abc^	0.83 ^a^	0.83 ^b^	0.24 ^a^	0.18 ^a^	186.4 ^ab^	280.7 ^cde^
	UVE17 D+Se	***5.80 ^de^***	***6.94 ^e^***	5.40 ^ab^	9.97 ^c^	0.83 ^a^	0.84 ^b^	0.25 ^a^	0.18 ^a^	***472.7 ^cd^***	354.3 ^ef^

The values represent the mean ± SE (n = 3). Fischer’s LSD post hoc test was used to determine the significance of differences between the averages (*p* < 0.05). The names of parameters that showed significance (*p* ≤ 0.05) for Selenium × Method × Cultivar interaction are indicated in **bold**. Treatments with different letters (within the columns) are significantly different. If treatments share one or more letters, they are not statistically different. Means in **bold and *italics*** show significant differences between the control and selenium treatment within a cultivar and application method. UVE14: drought-tolerant cultivar, UVE17: drought-susceptible cultivar, flow: flowering stage, pod: pod-filling stage, D: drought stress, D+Se: drought stress under selenium treatment, Fv/Fm: ratio of variable fluorescence to maximum fluorescence, PI_abs_: performance index absorbance, PI_total_: total performance index, DI_0_/RC: flux of energy dissipated (heat) per reaction centre (RC), SC: stomatal conductance.

**Table 4:** Mean photosynthetic pigments’ content in UVE14 and UVE17 under drought stress with different selenium concentrations and application methods at flowering and pod-filling stages.

	Treatment	Chl *a* (mg/ g FW)		Chl *b* (mg/ g FW)		Tot Chl (mg/ g FW)	
Method		Flow	Pod	Flow	Pod	Flow	Pod
	UVE14D	1.25 ^c^	1.08 ^a^	0.62 ^d^	0.46 ^a^	1.99 ^e^	1.65 ^efg^
	UVE14D+Se	***0.96 ^ab^***	0.79 ^a^	***0.41 ^abc^***	0.47 ^a^	***1.42 ^abc^***	***1.33 ^bcd^***
Seed	UVE17D	0.92 ^a^	0.82 ^a^	0.44 ^abc^	0.35 ^a^	1.15 ^a^	1.18 ^bc^
	UVE17D+Se	1.12 ^abc^	1.65 ^a^	0.53 ^bcd^	0.92 ^a^	***1.84 ^de^***	***0.89 ^a^***
	UVE14D	1.22 ^bc^	1.15 ^a^	0.54 ^cd^	0.52 ^a^	1.60 ^bcd^	1.11 ^bc^
	UVE14D+Se	1.13 ^abc^	0.96 ^a^	0.53 ^bcd^	0.49 ^a^	1.24 ^ab^	***1.79 ^fg^***
Foliar	UVE17D	1.16 ^abc^	1.04 ^a^	0.33 ^a^	0.38 ^a^	1.46 ^abc^	1.18 ^bc^
	UVE17D+Se	1.03 ^abc^	0.84 ^a^	0.38 ^ab^	0.51 ^a^	1.28 ^ab^	***1.51 ^def^***
	UVE14D	1.15 ^abc^	1.08 ^a^	0.60 ^d^	0.45 ^a^	1.74 ^cde^	1.44 ^cde^
	UVE14D+Se	1.14 ^abc^	1.20 ^a^	0.52 ^bcd^	0.66 ^a^	1.53 ^bcd^	***1.86 ^g^***
Soil	UVE17D	0.98 ^abc^	0.84 ^a^	0.61 ^d^	0.42 ^a^	1.73 ^cde^	1.06 ^ab^
	UVE17D+Se	0.95 ^ab^	1.16 ^a^	0.50 ^bcd^	0.67 ^a^	1.42 ^abc^	***1.55 ^def^***

The values represent the mean ± SE (n = 3). Fischer’s LSD post hoc test was used to determine the significance of differences between the averages (*p* < 0.05). The names of parameters that showed significance (*p* ≤ 0.05) for Selenium × Method × Cultivarinteraction are indicated in bold. Treatments with different letters (within the columns) are significantly different. If treatments share one or more letters, they are not statistically different. Means in **bold and *italics*** show significant differences between the control and selenium treatment within a cultivar and application method. UVE14: drought-tolerant, UVE17: drought-susceptible, flow: flowering stage, pod: pod-filling stage, D: drought stress, D+Se: drought stress under selenium treatment, Chl *a*: chlorophyll *a*, Chl *b*: chlorophyll *b*, Tot Chl: total chlorophyll, FW: fresh weight.

**Table 5:** Analysis of variance (ANOVA) showing the main effects and interactions of cultivar, selenium concentration, and application method on growth and yield parameters of vegetable soybean.

	Selenium (Se)	Method (M)	Cultivar (C)	Se *×* M	Se *×* M *×* C
BPP	1.778	1.333	3.361 *	3.44 ***	1.694
PH	111.42 **	94.97 **	89.197 **	66.901 *	131.346 ***
PPP	3.674	21.30 ***	79.51 ***	5.299 ***	1.937
SPP	0.111	7.38 ***	20.25 ***	2.299	12.06 ***

*p ≤ 0.05, **p ≤ 0.01, ***p ≤ 0.001. Asterisks indicate the level of significance. BPP: number of branches per plant, PH: plant height, PPP: number of pods per plant, SPP: number of seeds per plant. Values represent the mean squares.

**Table 6:** Fishers LSD post hoc test representing the effect of selenium application methods on the mean values of the morphological features of drought-stressed edamame.

Selenium Concentration	Method	BPP	PPP
0 mg/L	Seed	6.5 ^b^	8.17 ^ab^
50 mg/L		6.5 ^b^	7.33 ^a^
0 mg/L	Foliar	5.33 ^a^	8.17 ^ab^
50 mg/L		7.00 ^b^	9.17 ^bc^
0 mg/L	Soil	7.00 ^b^	9.50 ^c^
50 mg/L		6.67 ^b^	11.25 ^d^

The values represent the mean ± SE (n = 3). Fisher’s LSD post hoc test was used to determine the significance of differences between the averages (*p* < 0.05). Treatments with different letters (within the columns) are significantly different. If treatments share one or more letters, they are not statistically different. BPP = number of branches per plant, PPP = number of pods per plant.

**Table 7:** Mean growth and yield responses of UVE14 and UVE17 under drought stress across selenium concentrations and application methods.

PH (cm)	Seed	Foliar	Soil
UVE14D	85.00 ^d^	74.22 ^cd^	67.77 ^abc^
UVE14D+Se	***70.00 ^ac^***	***59.44 ^a^***	61.22 ^ab^
UVE17D	66.44 ^abc^	64.33 ^abc^	82.22 ^d^
UVE17D+Se	71.00 ^bc^	70.22 ^abc^	***62.56 ^ab^***
SPP	Seed	Foliar	Soil
UVE14D	10.00 ^bc^	8.50 ^ab^	13.500 ^d^
UVE14D+Se	10.00 ^bc^	8.50 ^ab^	***10.33 ^c^***
UVE17D	7.00 ^a^	10.00 ^bc^	7.00 ^a^
UVE17D+Se	***9.00 ^bc^***	8.50 ^ab^	***10.33 ^c^***
PPP	Seed	Foliar	Soil
UVE14D	8.67 ^cd^	7.33 ^abc^	7.00 ^ab^
UVE14D+Se	7.33 ^abc^	6.33 ^a^	8.00 ^bcd^
UVE17D	7.67 ^abcd^	9.00 ^d^	12.00 ^e^
UVE17D+Se	7.33 ^abc^	***12.00 ^e^***	***14.50 ^f^***
BPP	Seed	Foliar	Soil
UVE14D	6.67 ^abc^	5.67 ^ab^	6.50 ^bcd^
UVE14D+Se	6.00 ^c^	6.00 ^abc^	6.33 ^bcd^
UVE17D	6.33 ^bcd^	5.00 ^a^	7.50 ^de^
UVE17D+Se	7.00 ^cde^	***8.00 ^e^***	7.00 ^cde^

The values represent the mean ± SE (n = 3). Fischer’s LSD post hoc test was used to determine the significance of differences between the means (*p* < 0.05). The names of parameters that showed significance (*p* ≤ 0.05) for Selenium × Method × Cultivarinteraction are indicated in **bold**. Treatments with different letters (within the columns and rows, per parameter) are significantly different. If treatments share one or more letters, they are not statistically different. Means in **bold and *italics*** show significant differences between the control and selenium treatment within a cultivar and application method. UVE14: drought-tolerant, UVE17: drought-susceptible, D: drought stress, D+Se: drought stress under selenium treatment. PPP = number of pods per plant, SPP = number of seeds per plant, BPP = number of branches per plant, PH = plant height. UVE14 = drought-tolerant, UVE17 = drought-susceptible.

## Data Availability

Data are contained within the article or Supplementary Materials.

## Supplementary Materials

The following supporting information can be downloaded: https://www.mdpi.com/article/10.3390/plants14213261/s1, Table S1: Correlation coefficient analysis representing the relationships between physio-chemical parameters (flowering and pod-filling stage) and the growth and yield parameters (R8 stage) of drought-stressed vegetable soybean under selenium soil-drench application; Table S2: Correlation coefficient analysis showing the relationships between physio-chemical parameters (flowering and pod-filling stage) and the growth yield parameters (R8 stage) of drought-stressed vegetable soybean grown under selenium as a foliar application; Table S3: Correlation coefficient analysis showing the relationships between physio-chemical parameters (flowering and pod-filling stage) and the growth and yield parameters (R8 stage) of drought-stressed vegetable soybean grown under selenium as a seed priming application.
