# Supplementary material for: Effectiveness of Soil, Foliar, and Seed Selenium Applications in Modulating Physio-Biochemical, and Yield Responses to Drought Stress in Vegetable Soybean (Glycine max L. Merrill)
Source: Plants (Basel). 2025 Oct 24;14(21):3261. doi: 10.3390/plants14213261 (PMC12609595; doi:10.3390/plants14213261)
Supplement: Supplementary file 1 [file plants-14-03261-s001.zip › plants-3895460-supplementary.pdf]

Supplementary Material

Table S1.1: Correlation coefficient analysis representing the relationships between physio-chemical parameters (flowering stage) and the growth and yield parameters (R8 stage) of drought-stressed edamame under selenium soil-drench application.

	PPP	SPP	SMPP	BPP	PH
**Fv/Fm**	-0.16	-0.23	-0.03	0.11	0.15
**PI_{total}**	0.07	-0.07	-0.23	-0.27	-0.38
**PI_{abs}**	-0.19	-0.10	-0.16	-0.02	-0.30
**DI_0/RC**	0.19	0.34	0.07	0.01	0.05
**ABS/RC**	0.17	0.35	0.08	0.05	0.12
**ET_0/RC**	0.08	0.43	-0.13	0.05	-0.15
**RWC**	**0.83***	-0.26	-0.31	0.22	-0.03
**Chl *a***	-0.44	0.39	0.65	-0.39	-0.39
**Chl *b***	-0.35	-0.04	0.14	0.19	0.31
**Tot Chl**	-0.41	-0.04	0.49	0.03	0.30
**g_s**	0.04	0.58	-0.07	0.02	0.14
**Car**	-0.09	-0.07	0.38	0.36	0.35
**APX**	-0.45	0.37	0.10	-0.39	-0.25
**GPX**	-0.10	-0.61	0.03	0.40	0.30
**SOD**	**0.85****	-0.30	-0.53	0.39	0.20
**EL**	0.21	0.47	-0.28	0.19	-0.12
**H_2O_2**	0.02	0.58	0.16	0.14	-0.06

*$p \leq 0.01$, **$p \leq 0.001$. Asterix indicates the level of significance. Fv/Fm = maximum photosystem II (PSII) quantum yield ratio of variable to maximum fluorescence, ABS/RC = energy absorbed per reaction centre, DI_0/RC = energy dissipated as heat per reaction centre. TRo/RC = trapped energy per reaction centre, ETo/RC = the flux of electrons transferred from quinone (QA) to plastoquinone (PQ) per active PSII per reaction centre, PI_{abs} = performance index absorbance, PI_{total} = total performance index, RWC = relative water content, Chl *a* = chlorophyll a, Chl *b* = chlorophyll b, Tot chl = total chlorophyll content, APX = ascorbate peroxidase, GPX = guaiacol peroxidase, SOD = superoxide dismutase, EL = electrolyte leakage, H_2O_2 = hydrogen peroxidase, PPP = number of pods per plant, SPP = number of seeds per plant, SMPP = Total seed mass per plant, BPP = number of branches per plant, PH =plant height.

Table S1.2: Correlation coefficient analysis representing the relationships between physio-chemical parameters (pod filling stage) and the growth and yield parameters (R8 stage) of drought-stressed edamame under selenium soil-drench application.

	PPP	SPP	SMPP	BPP	PH
**Fv/Fm**	0.13	0.04	-0.34	0.03	0.19
**PI_{total}**	0.47	-0.35	-0.37	0.16	0.29
**PI_{abs}**	0.64	0.02	-0.71	0.32	0.04
**DI_0 /RC**	0.22	-0.43	-0.17	0.08	0.01
**ABS/RC**	-0.05	-0.15	0.08	0.00	0.00
**ETR/RC**	-0.27	0.05	0.30	-0.54	0.56
**RWC**	0.77	-0.11	-0.57	0.17	-0.11
**Chl *a***	-0.15	0.31	0.37	-0.49	0.08
**Chl *b***	0.06	-0.08	-0.19	-0.16	0.30
**Tot Chl**	-0.33	0.30	0.35	-0.35	0.26
**g_s**	0.47	0.18	-0.35	0.04	-0.37
**Car**	0.13	0.05	0.18	-0.14	0.26
**APX**	-0.28	-0.12	0.24	-0.29	0.44
**GPX**	-0.43	-0.10	0.21	-0.48	0.61
**SOD**	-0.12	-0.45	0.04	0.44	-0.08
**EL**	0.23	-0.04	0.34	-0.14	-0.51
**H_2O_2**	-0.11	-0.18	0.05	-0.43	0.36

*$p \leq 0.01$,**$p \leq 0.001$. Asterix indicates the level of significance.Fv/Fm = maximum photosystem II (PSII) quantum yield ratio of variable to maximum fluorescence, ABS/RC = energy absorbed per reaction centre, DI_0/RC = energy dissipated as heat per reaction centre. TRo/RC = trapped energy per reaction centre, ETo/RC = the flux of electrons transferred from quinone (QA) to plastoquinone (PQ) per active PSII per reaction centre, PI_{abs} = performance index absorbance PI_{abs} = performance index absorbance, PI_{total} = total performance index, DI_0/ RC = flux of energy as dissipated (heat) per reaction centre (RC), Chl *a* = chlorophyll *a*, Chl *b* = chlorophyll *b*, Tot Chl = total chlorophyll, APX = ascorbate peroxidase, GPX = guaiacol peroxidase, SOD = superoxide dismutase, EL = electrolyte leakage, H_2O_2 = hydrogen peroxidase, PPP = number of pods per plant, SPP = number of seeds per plant, SMPP = Total seed mass per plant, BPP = number of branches per plant, PH =plant height.

Table S2.1: Correlation coefficient analysis showing the relationships between physio-chemical parameters (flowering stage) and the growth yield parameters (R8 stage) of drought-stressed edamame grown under selenium as a foliar application

	PPP	SPP	SMPP	BPP	PH
**Fv/Fm**	-0.06	0.21	-0.64	-0.18	0.54
**PI_{total}**	-0.41	-0.18	0.01	0.08	-0.17
**PI_{abs}**	-0.43	-0.20	-0.33	-0.29	-0.03
**DI_0/RC**	0.48	-0.02	0.26	0.28	0.06
**ABS/RC**	0.46	0.06	0.16	0.18	0.21
**ET_0/RC**	0.34	-0.05	0.11	0.09	0.22
**RWC**	-0.34	-0.46	0.36	0.15	-0.15
**Chl *a***	-0.20	0.32	0.50	-0.22	0.34
**Chl *b***	-0.42	-0.35	0.72	0.41	-0.02
**Tot Chl**	-0.07	0.24	0.65	-0.03	-0.51
**g_s**	0.67	0.33	-0.16	0.55	0.00
**Car**	-0.69	0.46	0.32	-0.68	0.01
**APX**	**-0.77***	-0.28	-0.01	-0.47	0.41
**GPX**	-0.45	-0.22	0.30	-0.22	-0.23
**SOD**	0.57	0.15	-0.25	0.37	-0.29
**EL**	-0.74	-0.47	0.21	-0.19	0.04
**H_2O_2**	-0.53	-0.33	-0.17	-0.07	0.37

*$p \leq 0.01$,**$p \leq 0.001$. Asterix indicates the level of significance. Fv/Fm = maximum photosystem II (PSII) quantum yield ratio of variable to maximum fluorescence, ABS/RC = energy absorbed per reaction centre, DI_0/RC = energy dissipated as heat per reaction centre. TRo/RC = trapped energy per reaction centre, ETo/RC = the flux of electrons transferred from quinone (QA) to plastoquinone (PQ) per active PSII per reaction centre PI_{abs} = performance index absorbance, PI_{total} = total performance index, DI_0/ RC = flux of energy as dissipated (heat) per reaction centre (RC), Chl *a* = chlorophyll *a*, Chl *b* = chlorophyll *b*, Tot Chl = total chlorophyll, APX = ascorbate peroxidase, GPX = guaiacol peroxidase, SOD = superoxide dismutase, EL = electrolyte leakage, H_2O_2 = hydrogen peroxidase, PPP = number of pods per plant, SPP = number of seeds per plant, SMPP = Total seed mass per plant, BPP = number of branches per plant, PH =plant height.

Table S2.2. Correlation coefficient analysis showing the relationships between physio-chemical parameters (pod filling stage) and the growth yield parameters (R8 stage) of drought-stressed edamame grown under selenium as a foliar application.

	PPP	SPP	SMPP	BPP	PH
**Fv/Fm**	-0.07	-0.73	-0.45	0.12	-0.09
**PI_{total}**	0.42	-0.07	-0.55	0.06	-0.09
**PI_{abs}**	-0.43	-0.20	-0.33	-0.29	-0.78
**DI_0/RC**	0.03	0.72	0.47	-0.07	0.06
**ABS/RC**	0.01	0.66	0.50	-0.03	0.07
**ET_0/RC**	0.30	0.50	0.19	-0.13	-0.14
**RWC**	-0.59	0.22	0.32	**-0.76***	0.14
**Chl *a***	-0.54	0.09	0.53	-0.30	0.18
**Chl *b***	0.13	-0.31	-0.30	0.04	-0.08
**Tot Chl**	-0.16	-0.44	-0.20	0.39	-0.16
**g_s**	0.73	0.09	-0.13	0.48	-0.10
**Car**	-0.61	-0.44	0.12	-0.16	-0.33
**APX**	-0.04	-0.52	-0.29	0.45	-0.14
**GPX**	0.74	0.52	-0.26	0.15	-0.01
**SOD**	**0.77***	0.03	-0.39	0.56	-0.12
**EL**	-0.08	-0.39	0.27	0.02	0.22
**H_2O_2**	-0.46	-0.33	0.60	0.30	0.15

*p ≤ 0.01,**p ≤ 0.001. Asterix indicates the level of significance. Fv/Fm = maximum photosystem II (PSII) quantum yield ratio of variable to maximum fluorescence, ABS/RC = energy absorbed per reaction centre, DI_0/RC = energy dissipated as heat per reaction centre. TRo/RC = trapped energy per reaction centre, ETo/RC = the flux of electrons transferred from quinone (QA) to plastoquinone (PQ) per active PSII per reaction centre.PI_{abs} = performance index absorbance, PI_{total} = total performance index, DI_0/ RC = flux of energy as dissipated (heat) per reaction centre (RC), Chl *a* = chlorophyll *a*, Chl *b* = chlorophyll *b*, Tot Chl = total chlorophyll, APX = ascorbate peroxidase, GPX = guaiacol peroxidase, SOD = superoxide dismutase, EL = electrolyte leakage, H_2O_2 = hydrogen peroxidase, PPP = number of pods per plant, SPP = number of seeds per plant, SMPP = Total seed mass per plant, BPP = number of branches per plant, PH =plant height.

.

Table S3.1. Correlation coefficient analysis showing the relationships between physio-chemical parameters (flowering stage) and the growth and yield parameters (R8 stage) of drought-stressed edamame grown under selenium as a seed priming application.

	**PPP**	**SPP**	**SMPP**	**BPP**	**PH**
**PI_{total}**	-0.46	0.04	0.18	-0.41	0.21
**PI_{abs}**	**-0.76***	0.00	-0.10	-0.24	0.33
**DIo/RC**	-0.01	0.28	0.18	0.26	-0.18
**ABS/RC**	0.38	-0.39	-0.15	-0.14	-0.07
**ET_0/RC**	-0.08	-0.11	-0.03	-0.22	-0.03
**RWC**	0.22	0.22	0.50	-0.39	-0.04
**Chl *a***	0.29	0.40	0.39	0.47	0.10
**Chl *b***	0.37	0.35	0.39	0.44	0.12
**Tot Chl**	0.38	0.52	0.41	0.23	0.28
**g_s**	0.11	-0.22	-0.30	0.53	0.12
**Caro**	0.27	0.64	0.57	0.34	0.23
**APX**	-0.40	-0.63	**-0.85****	0.02	-0.47
**GPX**	-0.22	-0.66	-0.31	-0.47	0.18
**SOD**	-0.02	-0.23	-0.44	0.64	-0.13
**EL**	-0.23	-0.33	-0.57	0.34	-0.06
**H_2O_2**	-0.18	0.15	-0.14	-0.35	-0.36

*$p \leq 0.01$,**$p \leq 0.001$. Asterix indicates the level of significance.Fv/Fm = maximum photosystem II (PSII) quantum yield ratio of variable to maximum fluorescence, ABS/RC = energy absorbed per reaction centre, DI_0/RC = energy dissipated as heat per reaction centre. TRo/RC = trapped energy per reaction centre, ETo/RC = the flux of electrons transferred from quinone (QA) to plastoquinone (PQ) per active PSII per reaction centre. PI_{abs} = performance index absorbance, PI_{total} = total performance index, DI_0/ RC = flux of energy as dissipated (heat) per reaction centre (RC), Chl *a* = chlorophyll *a*, Chl *b* = chlorophyll *b*, Tot Chl = total chlorophyll, APX = ascorbate peroxidase, GPX = guaiacol peroxidase, SOD = superoxide dismutase, EL = electrolyte leakage, H_2O_2 = hydrogen peroxidase, PPP = number of pods per plant, SPP = number of seeds per plant, SMPP = Total seed mass per plant, BPP = number of branches per plant, PH =plant height.

Table S3.2. Correlation coefficient analysis showing the relationships between physio-chemical parameters (pod filling stage) and the growth and yield parameters (R8 stage) of drought-stressed edamame grown under selenium as a seed priming application.

	**PPP**	**SPP**	**SMPP**	**BPP**	**PH**
**Fv/Fm**	0.57	-0.12	0.08	0.13	-0.14
**PI_{total}**	0.62	0.25	0.27	0.24	0.14
**PI_{abs}**	0.57	-0.44	-0.08	0.22	-0.35
**DI_0/RC**	-0.58	0.07	-0.13	-0.06	0.07
**ABS/RC**	-0.56	0.08	-0.13	-0.01	0.07
**ET_0/RC**	-0.15	0.36	0.18	0.14	0.25
**RWC**	-0.39	0.56	0.05	0.02	0.67
**Chl *a***	-0.09	0.06	-0.05	0.27	0.26
**Chl *b***	-0.11	0.08	-0.08	0.17	0.28
**Tot Chl**	0.56	0.41	0.72	-0.02	0.26
**g_s**	-0.41	0.02	-0.31	-0.27	0.09
**Car**	0.43	0.51	0.73	-0.09	0.17
**APX**	0.45	0.48	0.89	-0.21	0.17
**GPX**	0.22	-0.57	-0.14	-0.59	-0.58
**SOD**	-0.53	-0.08	-0.29	-0.34	-0.10
**EL**	-0.16	-0.55	-0.70	0.01	-0.45
**H_2O_2**	0.32	-0.13	0.09	0.38	-0.25

*$p \leq 0.01$,**$p \leq 0.001$. Asterix indicates the level of significance. Fv/Fm = maximum photosystem II (PSII) quantum yield ratio of variable to maximum fluorescence, ABS/RC = energy absorbed per reaction centre, DI_0/RC = energy dissipated as heat per reaction centre. TRo/RC = trapped energy per reaction centre, ETo/RC = the flux of electrons transferred from quinone (QA) to plastoquinone (PQ) per active PSII per reaction centrePI_{abs} = performance index absorbance, PI_{total} = total performance index, DI_0/ RC = flux of energy as dissipated (heat) per reaction centre (RC), Chl *a* = chlorophyll *a*, Chl *b* = chlorophyll *b*, Tot Chl = total chlorophyll, APX = ascorbate peroxidase, GPX = guaiacol peroxidase, SOD = superoxide dismutase, EL = electrolyte leakage, H_2O_2 = hydrogen peroxidase, PPP = number of pods per plant, SPP = number of seeds per plant, SMPP = Total seed mass per plant, BPP = number of branches per plant, PH =plant height.
